# Relationship of Ageing to Insulin Resistance and Atherosclerosis

**DOI:** 10.3390/metabo15090613

**Published:** 2025-09-15

**Authors:** Xiaoyu Hao, Siying Tu, Da Pan, Wang Liao, Ligang Yang, Shaokang Wang, Guiju Sun

**Affiliations:** 1 Key Laboratory of Environmental Medicine and Engineering of Ministry of Education, Department of Nutrition and Food Hygiene, School of Public Health, Southeast University, Nanjing 210009, China; haoxiaoyu1386@126.com (X.H.); 230248566@seu.edu.cn (S.T.); pan_da@seu.edu.cn (D.P.); wangliao@seu.edu.cn (W.L.); ligangyang@seu.edu.cn (L.Y.); gjsun@seu.edu.cn (G.S.); 2 Clinical Medical Research Center for Plateau Gastroenterological Disease of Xizang Autonomous Region, School of Medicine, Xizang Minzu University, Xianyang 712082, China

**Keywords:** ageing, insulin resistance, atherosclerosis, molecular mechanism, clinical application

## Abstract

Ageing drives a vicious cycle of insulin resistance (IR) and atherosclerosis through shared pathological pathways. This review aims to synthesise the current understanding of the molecular mechanisms that connect ageing, IR, and atherosclerosis, with a particular focus on oxidative stress, chronic inflammation, and metabolic disturbances. We systematically summarise evidence demonstrating how age-related mitochondrial dysfunction promotes IR, which in turn accelerates atherosclerotic progression. Based on this integration, we conclude that the intertwined nature of these processes reveals promising therapeutic targets. Targeting these shared pathways, such as with senolytic agents or anti-inflammatory agents, may offer novel strategic insights for concurrently mitigating IR and atherosclerosis in the ageing population.

## 1. Introduction

Ageing refers to the phenomenon in which the organism’s physiological and psychological adaptability to the environment decreases progressively and gradually leads to death. Ageing can be divided into two categories: physiological ageing and pathological ageing. The former refers to the process of physiological deterioration that occurs after maturity, while the latter is an age-related change caused by various external factors (including various diseases). Aging is accompanied by a gradual accumulation of cognitive and physical impairments, as well as an increased risk of developing several diseases, including pulmonary fibrosis, kidney disease, hepatic steatosis, obesity-related metabolic syndrome, type I and type II diabetes, cancer, diabetes mellitus, cardiovascular disease, and musculoskeletal and neurodegenerative diseases [1,2]. Current research suggests that ageing is the result of a combination of factors such as stem cell decline, DNA degeneration, dietary and psychiatric factors, and active ageing genes [3,4,5]. Since the application of experimental methods to study aging at the end of the 19th century, numerous doctrines on aging have been proposed, including the doctrine of somatic cell mutation, free radicals, natural cross-linking of biomolecules, immunology, telomere theory and neuroendocrine theory, etc., but a unified theory of aging has yet to be formed. In the new theory on ageing, it is explained that five factors cause ageing and ageing-induced death, the first of which is the harmful changes that occur in the cellular structure. The second factor is the cessation of adult cell division, resulting in an inability to restore cellular structure. The third factor is that cells do not die as a result of the accumulation of harmful changes that occur during the life of the organism, but rather as the accumulation of harmful changes increases, making the cells function worse. The fourth factor is the inability of stem cells to regenerate tissue by replacing it with new cells. The last factor is that when one of the vital functions of an organism ceases, all cells die suddenly, rather than gradually throughout their lifetimes [6].

In a study by López-Otín et al. [2], 12 hallmarks of ageing were proposed as genomic instability, telomere attrition, epigenetic alterations, loss of proteostasis, disabled macroautophagy, deregulated nutrient-sensing, mitochondrial dysfunction, cellular senescence, stem cell exhaustion, altered intercellular communication, chronic inflammation, and dysbiosis. The interplay of these hallmarks is complexly linked, and each is an important entry point for exploring the ageing process as well as developing new anti-ageing drugs.

Insulin is a protein hormone secreted by pancreatic beta cells in the pancreas, stimulated by endogenous or exogenous substances such as glucose, lactose, ribose, arginine, and glucagon [7]. Insulin is the only hormone in the body that lowers blood glucose while promoting glycogen, fat, and protein synthesis. It achieves this not only by promoting glucose processing in skeletal muscle and adipose tissue and inhibiting gluconeogenesis in the liver, but also by regulating nutrient delivery to target tissues through its action on the microvasculature [8,9]. Insulin resistance (IR), physiologically defined as a state of reduced responsiveness of insulin-targeted tissues to high physiologic insulin levels, is recognised as a causative driver of many modern diseases, including metabolic syndrome, nonalcoholic fatty liver disease, atherosclerosis, and type II diabetes [10]. The gradual and irreversible decline in the structure and function of multiple organ systems with advancing age can lead to the onset and progression of metabolic disorder-related diseases centred on IR.

Atherosclerosis is a chronic vascular inflammation mediated by traditional and non-traditional risk factors. It has traditionally been recognised as a cholesterol-accumulating disease caused by the retention of lipoproteins, including low-density lipoproteins (LDL), in the intima of the arteries [11,12]. The basic pathological change is the formation of plaques on the intimal surface of the arteries, with lipid streaks, fibrous plaques and atheromatous plaques. Continued aggravation of the lesion may result in secondary lesions such as calcification, atheromatous ulcer formation, thrombosis, and intraplaque haemorrhage. The aetiology and pathogenesis of atherosclerosis are complex and have not yet been fully elucidated. The main risk factors are hyperlipidemia, hypertension and heavy smoking, as well as diabetes mellitus, obesity, immune damage and genetic factors. Cardiovascular diseases are especially prevalent and detrimental among older people, contributing to high rates of morbidity, disability and mortality [13,14]. Research indicates that vascular ageing is also a significant risk factor for atherosclerotic cardiovascular disease, accelerating the progression of atherosclerosis [15].

As we age, the process of aging contributes to IR through mechanisms such as oxidative stress, inflammation, and metabolic disorders [16]. This, in turn, accelerates the formation of atherosclerosis by causing endothelial dysfunction, lipid abnormalities, and chronic inflammation. Additionally, vascular aging exacerbates systemic metabolic issues, creating a positive feedback loop among these factors. The molecular mechanisms linking aging, IR, and atherosclerosis involve pathophysiological processes at various levels. During aging, mitochondrial dysfunction leads to the accumulation of reactive oxygen species (ROS), which triggers oxidative stress. This stress impairs the insulin signaling pathway by inhibiting IRS-1/PI3K/Akt, thereby promoting IR [17]. At the same time, senescent cells secrete pro-inflammatory factors, such as IL-6 and TNF-α, which sustain chronic low-grade inflammation through the NF-κB and NLRP3 inflammasome pathways. This exacerbates IR and promotes the formation of atherosclerotic plaques.

Furthermore, IR increases the synthesis of very-low-density lipoprotein (VLDL) and decreases high-density lipoprotein (HDL) due to hyperinsulinemic and lipotoxic effects, which leads to lipid metabolism disorders and vascular endothelial dysfunction. Aging-related cell cycle arrest and telomere shortening also accelerate vascular aging and increase plaque instability [18]. These processes reinforce each other through three core mechanisms: oxidative stress, chronic inflammation, and metabolic disorders, forming a self-perpetuating pathological cycle. Targeting these common pathways may offer new therapeutic strategies to improve IR and atherosclerosis simultaneously.

This review attempts to explore the effects of ageing on IR as well as atherosclerosis, to discuss the association between IR and atherosclerosis, to summarise the results of the relevant research literature, and to draw reasonable and reliable conclusions.

## 2. Theories on the Mechanisms of Aging and Measures to Delay Aging

### 2.1. Mechanisms of Ageing Doctrine

Ageing is the gradual deterioration of the body’s functions over time, affecting the whole organism, tissues, cells, and even molecules. Research has revealed that the mechanisms of ageing are so complex that no single theory can fully explain them. As a result, many ageing-related doctrines have been proposed to explore the mechanisms of ageing from various levels. These theories consider the interactions among different components to provide both theoretical and experimental foundations for efforts to slow the ageing process.

#### 2.1.1. Somatic Cell Mutation Theory

The somatic cell mutation theory was first proposed by Szilard in 1959 [19,20], which combines environmental and genetic factors and has similarities to the free radical damage theory. The doctrine holds that somatic cells can undergo genetic mutations, and that the accumulation of mutated somatic cells to a certain extent can alter the structure and vitality of enzymes, preventing them from repairing damage and leading to chromosomal aberrations, which in turn can cause ageing and death of the organism. DNA repair mechanisms include base excision repair (BER), nucleotide excision repair (NER), and double-strand break repair (DSBR). However, the activity of mutation-associated repair enzymes, such as PARP1 and XRCC1, declines with age, resulting in the accumulation of mutations. These mutations can disrupt DNA methylation and histone modification patterns, which in turn can lead to altered gene expression.

How do somatic mutations influence the ageing process? Additionally, can the rate at which these mutations accumulate determine the lifespan of a species? Szilard proposed that the survival of somatic cells decreases at an accelerated rate as individuals age. He hypothesised that when the percentage of somatic cell survival reaches a specific threshold, the likelihood of that individual dying within a year approaches 100%. Based on this theory, he established a correlation between the percentage of somatic cell survival and the age at which an individual dies [20]. A subsequent study by Cagan et al. [21] on somatic mutation rates in mammals with increasing longevity showed that analysis of the landscape of somatic mutations in the intestinal crypts of 16 species of mammals using whole-genome sequencing revealed large differences in the number of somatic single-base substitutions across species and between individuals within each species. In a study involving five species—dogs, humans, mice, naked mole rats, and rats—linear regression analysis confirmed a significant accumulation of somatic mutations with age. This finding raises an important question: Are the differences in mutation accumulation related to longevity? Building on Cagan’s research, a follow-up study by Garger et al. [22] provided a crucial answer. This study surveyed eight phenotypic traits across 15 mammalian species and found that each trait was independently associated with lifespan. The strength of the correlation between each phenotypic trait and longevity was assessed, revealing that somatic mutation rate had the strongest association. This suggests that the somatic mutation rate is the most significant predictor of mammalian longevity.

**Figure 1:**
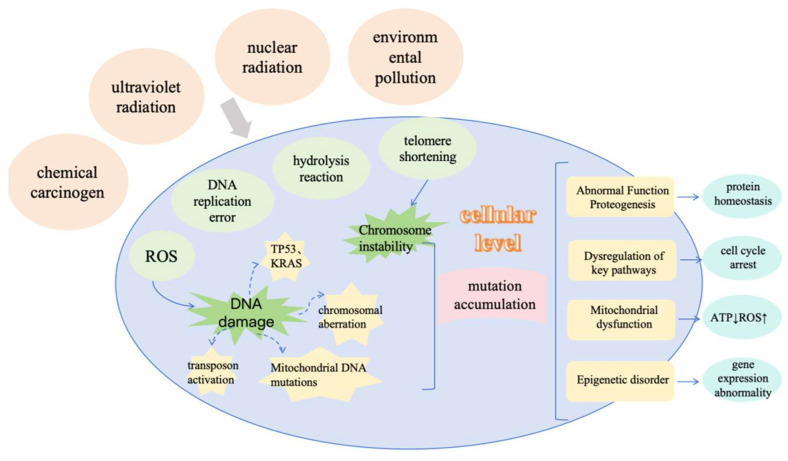
Mechanism of the somatic cell mutation doctrine Internal and external factors, such as nuclear radiation, environmental pollution, ultraviolet radiation, chemical carcinogens, reactive oxygen species (ROS), DNA replication errors, and hydrolysis reactions, can directly or indirectly trigger DNA damage and chromosome instability. This leads to cellular telomere shortening, mitochondrial DNA mutations, and transposon activation, resulting in the accumulation of mutations and abnormalities in the functioning of key genes such as TP53 and KRAS. Consequently, this accumulation manifests as epigenetic disorders, protein homeostasis imbalance, dysregulation of essential pathways, and mitochondrial dysfunction. Ultimately, these changes lead to cell-cycle arrest, abnormal gene expression, and mutations in normal cells. The upward and downward arrows indicate increase and decrease, respectively.

Although the conventional wisdom is that somatic mutations arise mainly from DNA replication errors, their rate of accumulation is positively correlated with the number of cell divisions. However, Abascal’s study challenges this. Abascal et al. [23] used nanosequencing technology (NanoSeq) to compare stem cells with differentiated cells and to study mutations in the absence of cell division. The results showed that in various somatic cell types, the rate of somatic mutation is largely independent of the rate of cell division, and that the rate of DNA mutation is the same in cells that do not divide and those that divide frequently. Previous studies have suggested that mutations can arise from two main sources: accidental misincorporation of nucleotides during DNA replication and DNA damage that occurs between replication cycles, which is not properly repaired. These studies introduced a model that connects the origin of mutations to the accumulation of cell divisions [24]. However, Abascal’s study suggests that the linear accumulation of somatic mutations in post-mitotic neurons has similar rates and characteristics to some mitotically active tissues. This key evidence suggests that the dominant mutation process can occur independently of cell division and may be driven by sustained endogenous DNA damage. This leads directly to the new question: if mutation accumulation is not dependent on cell division, can it still explain ageing? The clinical study by Robinson et al. [25] provides negative evidence for this. It was found that in some cancers, defective proofreading due to mutations in the structural domain of the acquired POLE/POLD1 nucleic acid exonuclease results in a significantly higher somatic mutation load with a unique mutational signature, but that in addition to an increased risk of cancer, individuals with germline POLE/POLD1 mutations do not exhibit the telltale features of premature senescence. These two studies challenge the universality of the somatic mutation doctrine and do not support the idea that ageing is due to widespread cellular dysfunction caused by somatic mutations that accumulate over time. Figure 1 provides a diagram illustrating the molecular mechanisms related to the somatic mutation doctrine [26].

#### 2.1.2. Free Radical Theory

The free radical theory of ageing was first proposed by Harman in 1965, which suggested that degenerative changes during ageing were due to the harmful effects of free radicals produced during normal cellular metabolism. Harman noted that free radicals produced during aerobic respiration lead to cumulative oxidative damage, resulting in cellular senescence and death, and pointed out the similarities between the effects of senescence and those of ionizing radiation, including mutagenesis, cancer, and severe cellular damage [27]. Over the past six decades, researchers have proposed several new variations on this concept, including the suggestion that mitochondria-derived reactive species play a central role and the proposal that the effectiveness of protein, lipid, and DNA repair systems shows a decreasing trend with age. These innovations have led to the widespread popularisation of the free radical theory of ageing [28].

The most common source of free radicals in biological systems is oxygen, and molecular oxygen (O_2_) is the basis for cellular metabolism and energy production [29]. The main free radicals produced in the body are superoxide anion radicals (O_2_•^−^), hydrogen peroxide (H_2_O_2_), hydroxyl radicals (·OH), and singlet oxygen (^1^O_2_), of which ^1^O_2_ is not a free radical but it can be oxidized and reduced to generate O_2_•^−^ to play a damaging role. Similarly, H_2_O_2_ is not a free radical. It is a non-radical reactive oxygen species that can be produced through the disproportionation of O_2_•^−^. H_2_O_2_ freely crosses cell membranes and is a critical redox signaling molecule.

Free radicals can extract hydrogen ions (H^+^) from polyunsaturated fatty acids (PUFAs) present in cell membrane phospholipids, creating lipid free radicals. These lipid free radicals then bind to O_2_ and undergo a process called peroxidation, leading to the production of lipid peroxides (LPO). These LPO decompose, triggering a series of reactions that produce malondialdehyde, a known mutagen. This chain of events can result in reduced membrane fluidity, mitochondrial dysfunction, and ultimately, apoptotic cell death. In addition, lipofuscin is a pigment that forms when LPO bind to proteins. This pigment accumulates in the internal organs and skin of ageing animals and humans. A significant buildup of lipofuscin in cells is considered an important indicator of ageing. At the same time, Free radicals can directly attack proteins. ROS can oxidise specific amino acid side chains in proteins, resulting in structural changes or loss of function. Additionally, ROS can cleave amino acid side chains and introduce carbonyl groups, altering the conformation of proteins. Carbonylated proteins are more likely to aggregate, which can contribute to diseases such as Alzheimer’s disease.

**Figure 2:**
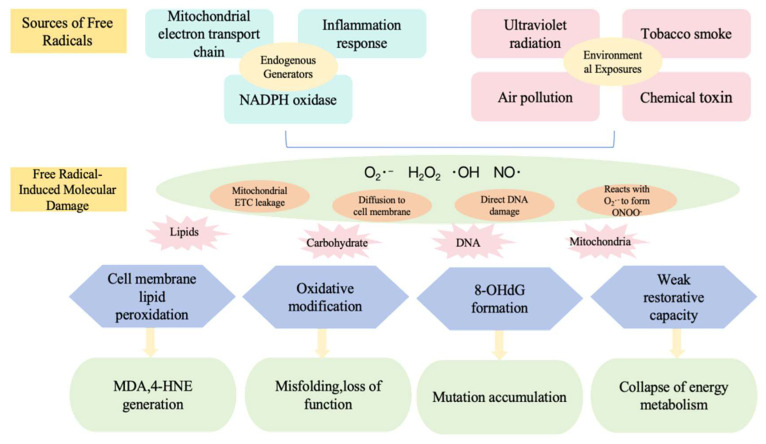
Diagram of the mechanism of the free radical doctrine of ageing Free radicals are produced through environmental exposures, such as ultraviolet light and tobacco smoke, as well as internal processes, including mitochondrial electron leakage and the activation of NADPH oxidase. These free radicals trigger lipid peroxidation, leading to the formation of malondialdehyde (MDA), DNA damage via the formation of peroxynitrite (ONOO-), and oxidative modifications of proteins. These detrimental effects ultimately result in mitochondrial dysfunction and genomic instability, which contribute to cellular aging and related diseases.

Oxidative stress is a powerful candidate mechanism for ageing, and it is the free radical theory of ageing that emphasises the effects of oxidative stress generated during normal metabolic processes on ageing. It is hypothesised that ageing is due to an increased accumulation of damage produced by ROS that is not controlled by antioxidant defence and cellular repair mechanisms with age. In this study, Robert et al. [30] assessed six different species of snakes, focusing on three main areas: animal physiology (measuring oxygen consumption), locomotor performance (as an indicator of health and survival), and cellular physiology (examining mitochondrial oxygen consumption and ROS production). They used reptiles as a proxy for their research and found that longer-lived species produced lower levels of ROS compared to shorter-lived species. This finding supports the free radical theory of aging at the cellular level. Similarly, Ali et al. [31] demonstrated that sex differences in free radical homeostasis during ageing are determinants of longevity, with females producing more free radicals in species where females have shorter lifespans than males. However, this theory has also faced criticism. For example, nude mole rats have a longer lifespan than ordinary rats, yet contrary to the expectations of the free radical theory of ageing, they exhibit higher levels of oxidative damage [32]. Despite some controversy over the free radical theory of ageing, the theory still provides a general theoretical framework for the study of ageing and facilitates the exploration of the mechanisms of ageing. Figure 2 presents a diagram illustrating the molecular mechanisms related to the free radical theory of aging [33].

#### 2.1.3. Immunological Theory

As we age, the immune system gradually declines. This age-related loss of immune cell regulation alters the production of both pro-inflammatory and anti-inflammatory mediators. The accumulation of senescent immune cells contributes to various diseases commonly associated with ageing, such as neurodegenerative diseases, cancer, cardiovascular diseases, and autoimmune disorders [34,35,36,37,38]. Under normal circumstances, the immune system does not react against the body’s tissues. However, various factors can cause the immune system to mistakenly identify certain self-tissues as foreign antigens, prompting an inappropriate immune response. These reactions can harm cells, tissues, and organs, which may lead to the onset of autoimmune diseases and can accelerate the ageing process and increase mortality.

With increasing age, bone marrow and thymus degenerate, peripheral lymphoid organs are damaged, and immune cell subpopulations are altered, resulting in weakened resistance to disease and decreased immune function [39]. Older people thus have increased autoimmunity and produce antibodies that do not discriminate between friend and foe, destroying their cells and accelerating the ageing process. In addition, as people age, the destruction and remodelling of immune organ structures, along with the development of innate and adaptive immune dysfunction, contribute to a higher likelihood of ineffective vaccinations. This increases susceptibility to infections, age-related diseases, and cancers in older individuals [39,40,41,42]. Overall, the function and adaptability of the immune system are key factors in organismal homeostasis and resistance to antigens. However, the current understanding of immune-related ageing mechanisms is still very limited and lacks biomarkers that serve as gold standards [43].

#### 2.1.4. Telomere Theory

The telomere theory of senescence suggests that cellular senescence is due to the shortening of the length of telomeres at the ends of chromosomes with age. Telomeres are non-coding repetitive DNA sequences at the ends of eukaryotic chromosomes and also contain proteins that bind to DNA [44]. When telomeres shorten to a critical length, known as the Hayflick limit, Shelterin proteins, such as TRF2, dissociate from the telomere. The exposed ends of the chromosomes are then recognised as DNA damage. Telomeres play a key role in regulating genome integrity, chromosome stability and other aspects of cellular physiology [45]. Telomeres shorten with each cell division, and once they reach a certain length, the cells can no longer divide. This issue arises because cells are unable to fully replicate their chromosomes during division due to dysfunctions in DNA polymerase. As a result, the last replicated DNA sequence may be lost, ultimately leading to cell death through a process called senescence. When telomeres become damaged, two key pathways are activated: the p53-p21 pathway and the p16-RB pathway. In the p53-p21 pathway, telomere damage activates the ATM/ATR kinase, which then phosphorylates p53 and increases the levels of p21, a cyclin-dependent kinase (CDK) inhibitor. This sequence of events leads to cell cycle arrest. In contrast, the p16-RB pathway responds to sustained stress by inhibiting CDK4/6 through the action of p16INK4a. This inhibition prevents the phosphorylation of the RB protein, blocks E2F transcription factors, and helps maintain the state of senescence.

Indeed, cells can compensate for missing portions of telomeres through the expression of telomerase. Telomerase is a ribonucleoprotein that exhibits reverse transcriptase activity. It consists of two main components: the reverse transcriptase (TERT) and an RNA template (TERC). This enzyme plays a crucial role in adding repetitive sequences to the ends of eukaryotic chromosomes, facilitating their replication. Additionally, telomerase is responsible for regulating and maintaining telomere length [46].

Telomeres are complexes of proteins and nucleotides comprising TTAGGG repeat sequences at the ends of eukaryotic chromosomes, and this base sequence is universal and consistent across most species. However, telomere lengths are species-specific, ranging from 4000 to 15,000 nucleotides [47]. In the vast majority of species, cellular senescence is associated with a reduction in telomere length. At the 3′ end of the telomere, there is no antiparallel strand, and each time it replicates, the telomere is shortened as a result because the DNA polymerase is unable to act at the 3′ end of the single strand. A meta-analysis on telomere length and all-cause mortality showed that telomere shortening was associated with increased all-cause mortality in the general population [48]. Similarly, in a meta-analysis by Ye et al. [49], it was concluded that telomere length decreases with actual age, although telomere length shows a nonlinear variation with actual age.

Currently, the sequencing of telomere length can effectively reveal relevant mechanisms regarding cancer and ageing. The study conducted by Schmidt et al. [50] used Telo-seq nanopore sequencing technology to sequence entire human telomeres effectively. This technique not only allowed for reproducible measurement of human cell volume and chromosome arm-specific telomere lengths but also reduced the rate of telomere shortening. Additionally, it enabled the analysis of allele-specific telomere lengths at a higher resolution. Given the application of these new technologies, can it be argued that patterns of telomere shortening are age-dependent based on sequenced telomere lengths? Sanchez’s team [51] conducted a longitudinal analysis involving 14 healthy individuals aged 18 to 77 years. They sequenced DNA from peripheral blood leukocytes of human donors and discovered a correlation between donor age and telomere lengths. Specifically, they found that the mean, median, first quartile, and third quartile lengths of telomeres were all affected by the donor’s age. Additionally, they observed that the mean and median telomere lengths in peripheral leukocytes decreased by approximately 27 base pairs each year. In the quantification of telomere length scores in a healthy ageing cohort, they found that the fraction of the distribution consisting of shorter telomeres increased with age. Telomeres will continue to play an important role in the study of ageing mechanisms as potential determinants of life expectancy and as distinct biomarkers of cellular senescence.

#### 2.1.5. Neuroendocrine Theory

The neuroendocrine doctrine holds that ageing is caused by hormone deficiency or defective target cell receptors. During the ageing process, the decline in the functioning of the neuroendocrine system parallels the ageing process, and the responsiveness of the body’s target cells to certain hormones or active substances is altered or markedly reduced with increasing age. Changes in the pancreas and thyroid are two of the more clinically significant changes in the ageing process. Approximately 40% of individuals between the ages of 65 and 74 years and 50% of individuals over the age of 80 years suffer from impaired glucose tolerance or diabetes, and between 5% and 10% of older women present with conditions such as decreased thyroxine and elevated thyrotropin [52]. Furthermore, the brain, as the central neuroendocrine system influencing age-related changes, specifically through the hypothalamus and pituitary gland, plays a crucial role in regulating endocrine secretion across the body. Brain cells produce a metabolic product called brown pigment, which is almost absent in the brains of newborn babies, but in the brains of people over 60 years old, brown pigment occupies more than half of the space inside the cells. This substance seriously affects the function of brain cells, reduces the regulation of the nervous system and accelerates the ageing process.

The senescence of endocrine glands is one of the more significant signs of the ageing process, and they age in a different chronological order. The decline of endocrine glands disrupts the endocrine hormone levels and affects each other [53]. Growth hormone secretion is closely linked to age. Research indicates that growth hormone peaks during mid-puberty, after which it declines by about 50% every 7 to 10 years. This decrease in growth hormone is also accompanied by a reduction in serum levels of insulin-like growth factor 1 (IGF-1) [54]. In response to these results, does the decline in growth hormone directly contribute to the symptoms of ageing? Can exogenous supplementation improve its function? In a study by Rosen et al. [55], it was found that growth hormone treatment resulted in increased energy and emotional stability in older adults. In addition, growth hormone enhances muscle strength, fat metabolism, bone density and skin thickness in older adults [56]. Another study found that treating middle-aged and older men with menopausal symptoms using growth hormone for up to six months led to significant improvements in their physical function, vasodilation, and psychosomatic symptoms compared to their pre-treatment state [57]. These findings strongly suggest a relationship between growth hormone deficiency and organic ageing, and that rational growth hormone supplementation can effectively alleviate or ameliorate some of the symptoms of ageing, thereby improving the quality of life in the older population. As another important hormone in the body, can a decrease in thyroid hormone also lead to the development of ageing-related symptoms? Research has shown that thyroid hormones play a central role in metabolism and immunity. Although hypothyroidism impairs metabolic rate, the reduction in thyroid hormones during ageing can be considered a form of reduced inflammation and can play a role in immune ageing. Therefore, maintaining normal thyroid function may help preserve the immune response in older adults [58]. These findings highlight the dual aspects of endocrine ageing: the necessity for targeted hormone replacement to sustain function, and the caution that excessive intervention may disrupt the natural adaptive mechanisms of ageing.

The effects of endocrine hormones such as growth hormone, thyroid hormone and sex hormones on the ageing process and related diseases and death are still being explored in depth. The downstream pathways and genetic determinants of these relevant hormones currently dominate ageing research, and genotyping the major functional factors of specific endocrine pathways may reveal the relationship between ageing and the emergence of specific outcomes [57]. The study of endocrine mechanisms related to ageing will be important for maintaining a healthy life and prolonging lifespan. Table 1 provides a summary of mechanistic doctrines related to aging, while Table 2 presents the related experimental studies [19,20,21,22,23,25,28,29,30,31,39,40,41,42,44,45,46,47,48,49,51,52,53,54,55,56,57].

### 2.2. Measures to Mitigate Ageing

As ageing is a complex biological process, it involves multiple mechanisms and pathways. Therefore, multiple therapeutic strategies need to be explored to slow down or even reverse the ageing process.

#### 2.2.1. Targeting Senescent Cells

Senescent cells are those that have ceased to divide but remain alive. They secrete pro-inflammatory factors known as SASPs, which contribute to a decline in tissue function. Therefore, removing these cells can slow down ageing to some extent. Currently, related studies develop transgenic INK-ATTAC mouse models that alleviate age-related phenotypes by selectively removing high p16-expressing cells from pregnant mice [59]. Selective removal of senescent cells is also possible through the use of a combination of dasatinib and quercetin, as well as BCL-2 family inhibitors [60].

Extracellular targets as well as immune-mediated clearance are also effective approaches to remove senescent cells. Characterisation of senescent cells reveals unique markers that act as senescence-associated autoantigens, and these can be used for immune system-mediated hemolytic activity and clearance. Senescent cells can also be removed by combining senescent antigens in a vaccinated manner. Senescent T-cell populations accumulate in obese adipose tissue and can lead to local and systemic inflammation [61]. Immunisation of senescent T cells through the use of CD153 peptide conjugates promotes the clearance of senescent T cells from adipose tissue and associated improvement in metabolic function.

#### 2.2.2. Delayed Telomere Shortening

Telomeres are protective caps at the ends of chromosomes that gradually shorten with cell division, eventually leading to cellular senescence. Maintaining telomere length may effectively slow down ageing through genetic interventions or the modulation of telomerase activity, which has been shown to reduce cellular ageing and enhance health in various model organisms [62]. Telomerase activation has gained prominence as a potential therapeutic approach for extending telomere length and subsequently prolonging cellular health. Common telomerase activators are TA-65 and Cycloastragenol (CAG). They can counteract telomere depletion due to cell division by activating telomerase, which catalyses the repetitive addition of TTAGGG nucleotides to chromosome ends. However, there are potential risks associated with this approach, and because it allows cells to proliferate uncontrollably, activation of telomerase may be associated with an increased risk of cancer [63].

Telomerase gene therapy is an emerging approach that attempts to address cellular ageing by directly regulating telomerase activity in cells. In related animal experiments, telomerase gene therapy using adeno-associated viral expression of TERT significantly improved health and reduced markers of ageing without increasing cancer incidence. However, telomerase gene therapy has not been tested in humans due to safety and ethical considerations.

#### 2.2.3. Metabolic Intervention

Ageing is closely related to many metabolic pathways, such as mTOR, AMPK, and insulin/IGF-1 signalling.

The main function of mTOR is to enhance anabolism and inhibit catabolism, and it has emerged as a potential target for inhibition in anti-ageing therapy. mTOR forms two complexes: mTOR complex 1 (mTORC1) and mTOR complex 2 (mTORC2). mTORC1 regulates cell growth and metabolism, whereas mTORC2 acts mainly as insulin/PI3K signaling [64]. mTOR is a major target for genetic control of ageing, and evidence from genetic studies supports the possibility that mTOR may be a major determinant of lifespan and ageing in yeast, flies, and mice [65]. Several studies have shown that the generalised reduction in mRNA translation caused by mTORC1 inhibition slows ageing [66]. Subsequent research has demonstrated that the mTOR inhibitor rapamycin effectively inhibits the SASP. Mass spectrometry analysis of SASP factors in senescent cells revealed that depleting mTOR reduced the secretion of approximately half of these factors by at least 20%. Similarly, inhibiting mTOR with rapamycin produced a comparable effect [67]. Postprandial increases in glucose and insulin activate mTORs in metabolic tissues, which regulate systemic metabolic homeostasis. In this context, mTORC2 plays a crucial role in glucose homeostasis through Akt. Akt activates mTORC1 through phosphorylation of TSC1/2. Activated mTORC1 phosphorylates IRS-1, leading to negative feedback regulation that blocks insulin signalling. mTORC1 and mTORC2 promote adipogenesis by regulating SREBP. mTORC1 enhances lipid storage while inhibiting lipolysis, β-oxidation, and ketogenesis. In addition, mTORC2 promotes glycogen synthesis and reduces gluconeogenesis [68].

AMPK is a stress kinase that maintains cellular homeostasis by participating in energy-producing pathways while inhibiting energy-consuming pathways to balance energy in the cell. It inhibits various cellular phenomena such as autophagy, tissue stress resistance, oxidative stress, endoplasmic reticulum stress, inhibition of inflammatory responses and ageing [69,70]. As AMPK activation decreases during ageing, it leads to the development of age-related complications such as cancer and other diseases [71]. The mechanisms underlying the decline and inactivation of AMPK are unclear, and they may be related to DNA damage and chronic inflammation disrupting AMPK signalling, which leads to a reduction in its expression. Epigenetic changes and mutations in the AMPK gene or upstream kinases such as LKB1 may also impair gene function and lead to reduced AMPK activity [72]. Metformin is a biguanide drug whose mechanism is to stimulate adenosine monophosphate to activate AMPK. A related study found that metformin extended the lifespan of roundworms by 50 per cent [73]. It exerts its life-extending effects primarily through the nutrient-sensing pathway. The nutrient-sensing pathway is a major pathway that regulates nutrient uptake and signalling, thereby affecting many metabolic processes and promoting growth. The insulin/IGF-1 signalling pathway consists of the INS/IGF-1 receptor and its adapter protein insulin receptor substrate 2 (IRS2), which is key to this process [74]. Metformin inhibits IGF-1 and IRS2 and prolongs lifespan [75]. In recent years, several studies have shown that metformin can prolong lifespan by modulating ROS production through the SIRT-3, Nrf-2/GPx7 and PRDX-2/SKN-1 signalling pathways [76,77,78]. In addition, it can extend lifespan by promoting autophagy-mediated components of attenuated catabolism and inhibiting the expression of mTOR-induced senescence-associated proteins.

Nicotinamide adenine dinucleotide (NAD+) is a classical coenzyme that mediates many redox reactions. NAD+ also plays an important role in the regulation of NAD+-depleting enzymes, including sirtuins, poly ADP-ribose polymerases (PARPs), and CD38/157 exoenzymes. NAD+ biosynthesis, particularly by nicotinamide phosphoribosyltransferase (NAMPT) and SIRT1-mediated, together regulate metabolism and circadian rhythms [79]. Sirtuins are a class of NAD+-dependent deacylating enzymes that play a key role in the regulation of cellular ageing, metabolism, DNA repair, inflammation, and stress resistance, and are often associated with longevity and healthy lifespan. Activation of sirtuins triggers a nuclear transcriptional program that enhances metabolic efficiency and upregulates mitochondrial oxidative metabolism and consequent anti-oxidative stress resistance [80]. It promotes this resistance by increasing antioxidant pathways as well as deacetylation or ADP-ribosylation of repair proteins to promote DNA damage repair [81]. Studies have shown that NAD+ levels and sirtuin activity decay with ageing [82]. A decrease in NAD+ was first detected in transgenic mice overexpressing SIRT1 in pancreatic β-cells. Mice exhibited enhanced glucose-stimulated insulin secretion when young but lost this phenotype in old age. When supplemented with NAD+, the metabolic phenotype was restored and insulin secretion was enhanced in older mice [83]. Meanwhile, NAD+ supplementation promoted the reactivation of sirtuins, prevented type II diabetes in wild-type mice due to diet and age-induced diabetes, and dramatically improved health and slowed ageing.

#### 2.2.4. Gut Microbiome Interventions

The gut microbiota is a complex community of microorganisms that reside in the human digestive tract, including bacteria, fungi, viruses, and archaea. These microorganisms play a crucial role in the ageing process of the human body. As we age, the composition and function of the microbiome change, which can impact the immune system, metabolic health, neurological function, and even longevity.

Inflammation is associated with a variety of diseases that develop with age, such as cardiovascular disease, metabolic syndrome, and chronic kidney disease. Inflammation associated with ageing may drive changes in the gut environment to some extent and changes in the microbiota. Key changes include the loss of microbial diversity, a reduction in beneficial symbiotic bacteria (such as Bifidobacteria and Lactobacilli), and an amplification of pro-inflammatory pathogens (such as Enterobacteriaceae). Related studies indicate that compared to healthy young adults, the gut microbiota of frail elderly individuals typically exhibits significant dysbiosis. Its overall characteristics include decreased microbial diversity, weakened microbial community stability, and disrupted functional communities. At the phylum level, a common trend is the shift from an altered Firmicutes-to-Bacteroidetes ratio (F/B ratio) [84]. As the ageing process progresses, changes in the gut microbiome become increasingly complex. These alterations may correlate with biological or functional age rather than chronological age. Gut microbial diversity inversely correlates with biological age, yet the co-abundance module comprising the genera *Ruminococcus*, *Coprobacillus*, and *Eggerthella* becomes more abundant with increasing biological age [85]. Additional studies indicate that the gut microbiota loses microbial diversity with advancing age, characterised by reduced numbers of glycolytic and proteolytic bacteria and increased subdominant species. In older adults, decreased abundance of bacteria, bifidobacteria, and thick-walled bacteria leads to chronic low-grade inflammation and diminished immune system function [86].

Studies of older adults and hyperagenarians who have reached the extremes of ageing have shown that microbiome characteristics are associated with elevated levels of inflammatory cytokines [87]. Nutrition and gut microbiota also have a significant impact on the development of cardiovascular disease. Rising obesity and diabetes are associated with disturbances in gut flora, IR and sedentary behaviours such as smoking, physical inactivity and poor nutrition, all of which are established risk variables for cardiovascular disease [88].

The association between gut microbiota and healthy ageing suggests that the microbiome may be a modifiable risk factor for health decline in older adults. Dietary interventions on the microbiome for related diseases are already available [89]. Regular consumption of a diet rich in fruits and vegetables may reduce the risk of related diseases in the older population. In addition, reports have shown that microecological modifiers such as probiotics and prebiotics are becoming increasingly important in preventing age-related imbalances in the intestinal microbiota and have a favourable anti-ageing effect. Probiotics help improve the health of the older people by increasing hypocholesterolemia and hypoglycemic effects, as well as preventing diarrheal diseases, improving inflammatory diseases, and preventing recurrent infections and colon cancer [90]. Promoting interactions between key components of the microbiota and the host epithelium through probiotic therapy modulates the transcriptional response of the gut microbiota [91]. Prebiotics are indigestible food components that selectively promote the growth of beneficial bacteria. Prebiotics stimulate bifidobacteria and lactobacilli, which help protect the immune system through the action of the microbiota [92]. Faecal microbiota transplantation is another way to restore the gut ecosystem. It involves transplanting faecal flora from a healthy donor into the patient’s gut to reestablish flora balance. The intestinal flora of ageing individuals typically shows a decrease in diversity, a decrease in beneficial bacteria, and an increase in pro-inflammatory bacteria. Faecal flora transplantation has been shown to improve metabolic function by reversing ageing-associated dysbiosis, modulating immune senescence, increasing short-chain fatty acids and modulating bile acid metabolism, thereby exerting an anti-ageing effect. Faecal flora transplantation has been widely attempted to treat intestinal diseases such as inflammatory bowel disease, constipation, irritable bowel syndrome, and colorectal tumours with favourable clinical outcomes [93].

#### 2.2.5. Lifestyle Interventions

Ageing is a complex biological process, but with scientific lifestyle interventions, it is possible to significantly slow down the progression of ageing and extend a healthy lifespan. Research has shown that a balanced diet, regular exercise, quality sleep, stress management and avoidance of risk factors such as smoking and alcohol abuse can work synergistically to reduce oxidative stress, inflammation and cellular damage and activate longevity-related pathways.

Diet is one of the key factors affecting the ageing process. By adjusting dietary structure, controlling caloric intake, and optimising nutritional combinations, we can activate longevity-related pathways, reduce oxidative damage, and delay cellular ageing. Common dietary interventions include calorie restriction, alternate-day fasting, time-restricted feeding, and abstinence from imitation diets [94].

Calorie restriction helps activate longevity-related pathways such as AMPK and SIRT1 to promote cellular repair. It also reduces free radical production and slows the decline of mitochondrial function. Related experimental animal studies have shown that calorie restriction delays brain ageing and subsequently increases insulin sensitivity. Calorie restriction is also effective in delaying sarcopenia, maintaining muscle mass, preventing age-related declines in physical activity, and reducing the metabolic costs of exercise that increase with age [95].

Intermittent fasting, a dietary pattern of cyclically alternating eating and fasting, has been extensively studied in recent years for its relationship to ageing metabolism. Unlike calorie restriction, intermittent fasting triggers metabolic shifts and cellular stress resistance through patterns of time-restricted eating, alternate-day fasting, or cyclical fasting to slow ageing. Studies have shown that intermittent fasting can have a wide range of effects on metabolic markers and risk factors or diseases, including body fat and blood pressure. In overweight individuals, consuming a diet of about 500 kcal with relatively high protein two days per week for six months led to a reduction in abdominal fat, lower blood pressure, and increased insulin sensitivity [96]. Intermittent diets and calorie restriction have similar effects on reducing visceral fat, insulin and IR. Intermittent diets have been found to prevent and cure insulin-related diseases in many animal studies. By fasting for 16 h per day, diabetic mice had reduced circulating levels of glucose, insulin, and leptin and normalised glucose tolerance. Intermittent fasting also ameliorates insulin deficiency and glucose intolerance in a rat model of type I diabetes through a mechanism involving pancreatic beta cell preservation [97]. However, in randomised controlled trials examining the effects of alternate-day fasting versus calorie-restricted diets or control diets on cardiometabolic risk factors, alternate-day fasting was found not to be superior to calorie restriction for weight loss, weight maintenance, and reduction in cardiometabolic risk factors [98].

The Mediterranean diet is recognised as one of the healthiest eating patterns in the world. Numerous studies have confirmed its effectiveness in reducing the risk of cardiovascular disease, diabetes, cognitive decline and some cancers, and in significantly increasing healthy life expectancy. The Mediterranean diet is characterised by a predominantly plant-based diet with a high intake of fruits and vegetables, whole grains instead of refined carbohydrates, as well as legumes and nuts more than three times a week. It is also recommended to consume moderate amounts of dairy products and eggs, and reduce the intake of meat foods. Related studies have shown that unsaturated fatty acids and polyphenols, which characterise the Mediterranean diet, are the basis for the prevention of cardiovascular disease, especially in vegetable-rich diets that show lower levels of total cholesterol and lower levels of systolic blood pressure. In older adults, the Mediterranean diet reduces impaired glucose oxidation and inflammatory responses compared with dietary patterns based on saturated fatty acids [99].

Sleep is one of the body’s most important repair mechanisms, and chronic sleep deprivation or poor quality can significantly accelerate the ageing process and affect longevity. Ageing is associated with a decrease in circadian rhythms of behaviour, including sleep. Older adults commonly exhibit decreased total sleep time, decreased sleep efficiency, increased sleep latency, increased nighttime awakenings, excessive daytime sleepiness, and increased daytime napping [100]. As we age, the circadian system undergoes major changes that affect behavioural rhythms, temperature regulation, and hormone release. Circadian rhythms provide organisms with an adaptive mechanism that coordinates cellular processes, physiological functions, and behaviours with the Earth’s predictable 24 h cycle of light and dark [101]. It is regulated by the “master biological clock” located in the suprachiasmatic nucleus (SCN) of the hypothalamus. The SCN synchronises internal biological signals with external light signals by transmitting time-of-day information through both synaptic and diffusible signals. It communicates this information to various clocks located in different regions of the brain as well as peripheral organs, including the heart, lungs, liver, and endocrine glands. This process helps align the timing of rhythmic activities throughout the body with the natural light/dark cycle [102]. The clocks of the peripheral organs further fine-tune the central clock-regulated food intake, energy expenditure, and systemic insulin sensitivity. For example, peripheral clocks in the gut regulate glucose absorption, peripheral clocks in muscle, adipose tissue, and liver regulate local insulin sensitivity, and peripheral clocks in the pancreas regulate insulin secretion. The development of IR may occur when there is a mismatch between different components of the circadian system and the daily rhythms of sleep–wake behaviour or food intake due to genetic, environmental, or behavioural factors.

Circadian rhythms deteriorate with age due to a number of factors. Compared to young SCN neurons, aged SCN neurons exhibited reduced inhibitory postsynaptic potentials [103]. Genetic, behavioural, and pathology-induced circadian disorders or arrhythmias have been shown to lead to a wide range of health consequences, including an increased risk of cancer, cardiovascular disease, obesity, immune disorders, infertility, and affective disorders [104].

Natural light is essential for the development of circadian rhythms to coordinate the physiology, metabolism and behaviour of most organisms. However, with the rapid development of science and technology, human beings are exposed to more and more high-energy blue light spectrum from artificial lighting. Blue light exposure is now a potential risk factor for the accumulation of retinal cellular damage and disruption of circadian rhythms. Related studies have shown that daily exposure to blue light affects a variety of phenotypes in Drosophila, including lifespan, neurodegeneration, mitochondrial physiology, energy metabolism, and neurotransmitter levels. The main effects of blue light on Drosophila observed in transcriptomics and metabolomics studies are related to neuronal function, including ageing and circadian rhythms [105]. When exposed to blue light for long periods of time at night, blue light can penetrate the cornea directly to the retina and induce ROS production, leading to impaired mitochondrial function. It also inhibits pineal melatonin secretion through the retino-hypothalamic pathway and interferes with the biological clock, especially through ipRGCs photosensitive cells, affecting their antioxidant, insulin-sensitising, and lipolytic effects. The results of animal experiments showed that chronic dim nocturnal artificial light exposure, especially blue light exposure, significantly increased hepatic lipid accumulation, dyslipidemia and imbalance of glucose homeostasis in obese or diabetic mice. The possible mechanism is to interfere with hepatic lipogenesis and lipolysis by inhibiting hepatic REV-ERB signalling [106]. At the same time, blue light activates NADPH oxidase and increases ROS, which in turn activate pro-inflammatory pathways such as NF-κB, accelerating ageing and metabolic abnormalities.

There are significant differences in the effects of blue light on different individuals, and some populations may be more sensitive to blue light due to genetic variants, manifesting as more intense photodamage, circadian rhythm disruption, or metabolic abnormalities. MTNR1B is a key regulator of melatonin receptor and blue light sensitivity, mainly expressed in the retina, pancreatic β-cells, adipose tissue, and brain, and plays a key role in circadian regulation, glucose metabolism, and insulin secretion. Variants in this gene are strongly associated with blue light sensitivity, sleep disorders and metabolic diseases. Related studies have shown that the G risk allele in MTNR1B rs10830963 is associated with higher MTNR1B expression, leading to enhanced inhibition of insulin secretion by melatonin, impaired insulin secretion, and increased risk of type II diabetes. It also causes an increase in fasting blood sugar levels, and higher blood sugar levels can directly contribute to the development of breast cancer [107]. Second, the G risk allele in rs10830963 was also associated with a later dim-light melatonin offset and a longer melatonin duration at night [108]. When MTNR1B is highly expressed, it may make individuals more sensitive to the melatonin-suppressing effects of blue light, which may lead to difficulty in falling asleep, decreased sleep quality, and delayed circadian rhythms.

Melatonin secretion is regulated by light, peaks at night, and directly affects the sleep–wake cycle, antioxidant defence, and immune regulation. Related studies have shown that melatonin production is reduced in patients with coronary artery disease. Melatonin administration (≤5 mg/day) was able to reduce hypertension [109] significantly. At the same time, melatonin also has antioxidant and metabolic regulation effects, can scavenge free radicals, protect mitochondrial function, reduce DNA damage, promote brown fat thermogenesis, and reduce the risk of obesity. Consequently, targeted interventions including melatonin supplementation, minimisation of blue light exposure, and circadian synchronisation could represent promising approaches to decelerate ageing processes and mitigate metabolic disease pathogenesis. Table 3 summarizes measures to delay ageing [59,60,62,65,66,67,68,69,70,71,72,73,74,75,76,77,78,79,80,81,82,83,90,91,92,93,94,95,96,97,98,99,100,101,102,103,104,105,106,107,108,109].

## 3. Insulin Resistance

IR is defined as a diminished response of body cells to insulin, resulting in reduced cellular utilisation of glucose, and it is a common pathological mechanism in the development of a variety of metabolism-related disorders. Insulin resistance is characterised by a diminished response to insulin stimulation in insulin-sensitive organs and tissues such as the liver, skeletal muscle, and adipose tissue. It is also described as a reduction in insulin sensitivity resulting from impaired insulin signalling, stemming from a weakened response to insulin signals regulating blood glucose levels [110]. The main causes of the disease include genetic factors, environmental factors, increased hormone production and other diseases.

### 3.1. Insulin Signaling Pathway

The insulin signalling pathway is an intracellular signalling pathway responsible for the metabolism of the organism, especially in growth and survival [111]. The process of the insulin signaling pathway involves several steps, starting with the binding of insulin and insulin-like growth factor to their respective receptors leading to receptor autophosphorylation, and binding of the phosphorylated receptor to its direct substrate leading to recruitment and phosphorylation of the receptor substrate, which in turn activates the downstream pathway [112]. The insulin receptor consists of two alpha subunits and two beta subunits. Insulin binding to the α-subunit induces a conformational change in the receptor that activates the tyrosine kinase activity of the β-subunit and triggers autophosphorylation. The Shc and IRS proteins activate two insulin signalling pathways: mitogenic signalling and metabolic signalling.

The central link between IRS proteins and the metabolic actions of insulin is through the PI3K/Akt signalling pathway. Recruitment and activation of PI3K is dependent on the binding of two SH2 structural domains in the regulatory subunit to tyrosine-phosphorylated IRS proteins [113]. The activation of the catalytic subunit leads to the rapid phosphorylation of phosphatidylinositol 4,5-bisphosphate (PIP2), resulting in the production of the lipid second messenger phosphatidylinositol (3,4,5)-trisphosphate (PIP3). This molecule recruits Akt to the plasma membrane, where it is phosphorylated to become active, subsequently initiating downstream signalling pathways [114]. Activated Akt plays a central regulatory role in insulin signalling by modulating the expression and activity of a variety of effector proteins, including metabolic enzymes, transcription factors, cell cycle regulatory proteins, and apoptosis-related proteins [115]. In metabolic regulation, Akt mediates the insulin effect through multifaceted mechanisms: (1) glucose transport, Akt phosphorylates AS160 protein to promote the transport of GLUT4 vesicles to the plasma membrane and enhance glucose uptake; (2) glycogen synthesis, Akt inhibits the activity of gluconeogenic synthase kinase 3 (GSK3) and releases its inhibitory effect on gluconeogenic synthase; (3) lipid metabolism (3) lipid metabolism, Akt up-regulates the expression of lipogenesis-related genes such as fatty acid synthase (FAS) through activation of sterol regulatory element-binding protein 1c (SREBP1c); (4) gluconeogenesis regulation, Akt inhibits gluconeogenesis-related genes such as phosphoenolpyruvate carboxykinase (PEPCK) and glucose-6-phosphatase (G6Pase) by phosphorylating the transcription factor FOXO1 and preventing its nuclear translocation in liver; (5) gluconeogenesis regulation. G6Pase) and other key enzymes of gluconeogenesis. These synergistic effects work together to maintain glucose homeostasis and energy metabolism balance in the body.

The second important branch of the insulin signalling pathway is the Grb2/SOS/Ras/MAPK pathway, which functions independently of PI3K/Akt. The SH3 structural domain at the amino-terminal end of Grb2 binds to the proline-rich region of the protein (SOS). SOS is the guanine nucleotide exchange factor (GEF) for Ras and catalyses the conversion of membrane-bound Ras from the inactive GDP-bound form (Ras-GDP) to the active GTP-bound form (Ras-GTP). Ras-GTP then interacts with and stimulates downstream effectors, which phosphorylate and activate MAPK to function [114].

**Figure 3:**
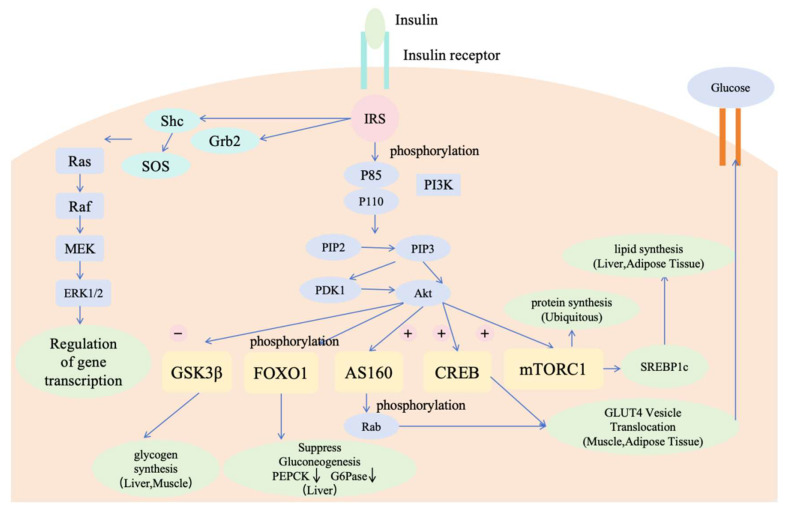
Diagram of insulin signaling pathway Upon insulin binding to the insulin receptor, the IRS-PI3K-Akt signaling cascade is activated through a series of phosphorylation events. This leads to downstream metabolic responses, including glycogen synthesis (via GSK3β inhibition) and GLUT4 translocation (mediated by AS160 and Rab). The pathway also regulates gene transcription through key transcription factors, including FOXO1 (phosphorylated and inactivated by Akt, resulting in the suppression of PEPCK expression) and CREB (activated to promote the expression of GLUT4 and other metabolic genes). Additionally, mTORC1 activation enhances the expression of genes involved in protein and lipid synthesis. The downward arrows indicate decrease.

The insulin signalling pathway is directly or indirectly associated with a wide range of diseases and is expressed in a variety of tissues in vivo. In recent years, it has been found that the insulin signalling pathway is not only one of the key signalling pathways of glucose metabolism, but also its factors are involved in the regulation of bone metabolism, hepatic IR, diabetic nephropathy, and vascular ageing. The insulin signalling pathway needs more in-depth research to discover more potential connections in order to solve more health problems. Figure 3 illustrates the mechanisms involved in the insulin signaling pathway [113,114,115].

### 3.2. Mechanisms of IR

Insulin resistance is typically characterised by compensatory hyperinsulinemia. In this state, insulin target organs—such as the liver, adipose tissue, and skeletal muscle—exhibit significantly diminished insulin responsiveness, thereby preventing the hormone from exerting its physiological effects on glucose regulation. Specifically, this manifests as uninhibited hepatic gluconeogenesis, enhanced lipolysis, and impaired glucose uptake and utilisation in skeletal muscle [116]. The physiological mechanisms behind IR are attributed to insufficient insulin action on target cells, which can occur for two main reasons: defects in the insulin receptors (such as reduced receptor expression) or issues with post-receptor signalling (impaired signalling pathways) [117].

The insulin receptor is a tyrosine kinase that binds specifically to insulin and plays a key role in insulin-mediated glucose homeostasis and cell growth [118,119]. Impaired insulin receptor binding is mainly characterised by a decrease in the affinity and number of target receptors on the cell membrane or structural abnormalities of target receptors that affect insulin-receptor binding [120]. One of the key mechanisms of IR involves abnormal phosphorylation and dysfunction of IRS proteins. In pathological states, serine/threonine phosphorylation (such as at Ser307 and Ser612 sites) replaces the normal pattern of tyrosine phosphorylation, which is typically mediated by TNF-α and free fatty acids through the JNK/IKKβ pathway. This shift can significantly inhibit the activity of IRS proteins. Additionally, proteins from the suppressor of cytokine signaling (SOCS) family, particularly SOCS3, promote the degradation of IRS-1 and IRS-2 via the ubiquitin-proteasome pathway. Chronic inflammation, characterised by elevated levels of IL-6, and overnutrition, such as consuming a high-fat diet, can inhibit the transcription of IRS genes. This inhibition results in decreased expression of IRS proteins. When the insulin receptor is not functioning properly, it is unable to recruit and phosphorylate downstream substrate proteins effectively. Since IRS proteins play a crucial role in insulin signalling, their dysfunction directly impairs the activation of downstream pathways, such as the PI3K/Akt pathway, ultimately leading to IR.

Insulin acts by binding to the insulin receptor and activating downstream signalling pathways. Impaired regulation of any one of the links in each of the pathways will affect the activation of the entire pathway to varying degrees. Among the two signalling pathways of insulin, the PI3K/AKT pathway plays a role in the regulation of metabolism. In contrast, the Grb2/SOS/Ras/MAPK pathway is mainly responsible for the control of cell growth and differentiation [121]. Impaired signalling in both pathways directly or indirectly leads to the development of IR.

The development of IR is influenced by multiple interconnected pathophysiological mechanisms, in addition to the abnormal function of insulin receptors and their substrates. One significant factor in this process is chronic low-grade inflammation. Conditions such as obesity and overnutrition can lead to the excessive secretion of pro-inflammatory factors, such as TNF-α and IL-6, from adipose tissue. These inflammatory factors play a role in phosphorylating the IRS-1 protein at the Ser307 site by activating the JNK (c-Jun N-terminal kinase) and IKKβ (IκB kinase) signalling pathways. This activation disrupts the normal tyrosine phosphorylation of IRS-1, ultimately impairing insulin signalling.

Abnormal metabolism of free fatty acids (FFAs) plays a significant role in IR. Elevated levels of circulating FFAs activate PKCθ, which induces the phosphorylation of serine residues on IRS-1, inhibiting the activation of the PI3K-AKT pathway. Additionally, excessive FFAs enter the mitochondria, leading to an overload of β-oxidation. This process triggers the accumulation of ROS, resulting in oxidative stress and further impairing insulin signalling. Furthermore, FFAs are metabolised into ceramides, which can activate PP2A, leading to the dephosphorylation of AKT. The accumulation of FFAs can also directly interfere with insulin signalling, worsening IR.

In addition, increased visceral fat is often closely associated with IR. In a state of IR, skeletal muscle and liver response to insulin is diminished, leading to decreased glucose uptake and increased gluconeogenesis in the liver, which in turn raises blood glucose levels. A number of genetic variants have also been associated with the risk of IR, and genetic factors may influence the distribution of fat, the ability to secrete insulin, and the sensitivity of cells to insulin. Abnormal secretion of hormones may also cause or aggravate IR to a certain extent, and lifestyle factors such as unhealthy diet, lack of exercise and sleep deprivation can also significantly affect insulin sensitivity. Overall, IR is the result of a multifactorial interaction involving endocrine, metabolic, genetic and environmental aspects.

### 3.3. IR Related Diseases

IR may cause a variety of diseases, not only elevating blood glucose and lipids, but also affecting nutrient metabolism. IR is the main pathologic basis of type II diabetes, and most patients with impaired glucose tolerance and type II diabetes have IR [122,123]. In addition, it has been shown that non-diabetic first-degree relatives of patients with type II diabetes may also be insulin resistant and that insulin predicts the development of significant type II diabetes [124,125]. When the action of insulin is diminished, the pancreas needs to secrete more insulin to maintain normal blood glucose levels. This may eventually lead to pancreatic failure, which in turn may lead to the development of diabetes. In addition, IR is one of the core symptoms of metabolic syndrome. Metabolic syndrome is a pathological condition in which the body’s metabolism of proteins, fats, carbohydrates and other substances is disturbed. It is a complex group of metabolic disorder syndromes, including IR, atherosclerosis, dyslipidemia, centripetal obesity, and hypertension. In 1988, Reaven suggested that IR is not only associated with type II diabetes but also with the aetiology of cardiovascular disease and pointed out that IR is accompanied by a series of abnormalities, which is described as metabolic syndrome [126]. The range of symptoms of metabolic syndrome is assessed using six indices: waist circumference, fasting blood glucose level, triglyceride level, HDL level, cholesterol level and blood pressure [127,128]. IR has different effects on the metabolic syndrome phenotype, and IR on the central nervous system may contribute to the development of obesity. Human appetite is tightly controlled by insulin action in the CNS, and data suggest that neuronal insulin signalling is necessary for weight control and glucose homeostasis [129]. IR in the liver may trigger the development of hyperglycemia. The absence of insulin receptors in the liver blocks the induction of insulin or feeding on Akt and Foxo1 phosphorylation and leads to unrestricted gluconeogenesis for hepatic glucose production, which triggers hyperglycemia [130,131]. In addition, cardiac IR carries the risk of triggering heart failure. It has been found that the absence of cardiac insulin receptor reduces cardiac Akt and Foxo1 phosphorylation and leads to heart failure and death in male mice at 7 to 8 weeks of age [132]. As well as IR occurring in skeletal muscle may disrupt glucose homeostasis and impair lifespan. When IR occurs in the vascular endothelium, it may cause hypertension.

IR is also associated with an increased risk of cardiovascular diseases such as atherosclerosis, heart disease and stroke. The occurrence of IR can lead to problems such as dyslipidemia and elevated blood pressure, which can aggravate the burden on the heart. In the cardiovascular system, insulin myocardial signalling, which controls metabolism and other biochemical aspects of cellular function, ensures cardiomyocyte function. Insulin binds to receptors on the membranes of cardiomyocytes, activating insulin signalling molecules, specifically the substrates of IRS-1/2 and PI3K/Akt. These molecules play a crucial role in stimulating the translocation of GLUT4 and promoting glucose metabolism. To generate sufficient ATP through glycolysis and oxidative phosphorylation, insulin also stimulates the exocytosis of GLUT4 and CD36. GLUT4 mediates glucose transport in the myocardium, whereas an increase in glucose flux from GLUT1 contributes to a decrease in the increase in GLUT4 exocytosis and an increase in GLUT4 concentration in the myocardium [133].

IR can lead to the accumulation of fat in the liver, which in turn causes non-alcoholic fatty liver disease. This may progress to hepatitis, liver fibrosis and even cirrhosis. In women, IR has been linked to polycystic ovary syndrome, which may lead to irregular periods, infertility, and other endocrine disorders. Other studies suggest that IR may be associated with an increased risk of neurodegenerative diseases such as Alzheimer’s disease. It may also be associated with an increased risk of certain types of cancer, such as breast and pancreatic cancer [134].

Overall, IR is a complex state of metabolic disorders that may lead to multiple health problems. Early recognition and intervention are therefore important. Improvement of lifestyle, such as a healthy diet, moderate exercise, and maintaining an ideal body weight, is often an effective way to manage IR.

### 3.4. The Relationship Between Aging and IR

Age-related dysfunction in insulin secretion may play a role in changes in glucose metabolism with age and may contribute to high rates of poor glucose tolerance in older populations. As ageing progresses, age-related increases in visceral adiposity and accumulation of senescent cells with inflammatory phenotypes lead to elevated levels of inflammatory cytokines. This interferes with insulin signalling, dysfunctioning the IRS-PI3K/Akt pathway, leading to impaired glucose uptake in muscle and adipocytes, reduced glycogen synthesis/storage in the liver, as well as failure to inhibit hepatic glucose production. Impaired absorption and storage of circulating lipids lead to elevated lipid levels, increased plasma levels of very low-density lipoproteins, and accelerated IR [135].

Mitochondria regulate many cellular processes and are key elements of cellular and organismal homeostasis. With ageing, mitochondrial function deteriorates due to a variety of intertwined mechanisms, including accumulation of mtDNA mutations, insufficient protein stabilisation leading to instability of the respiratory chain complex, reduced organelle turnover, and changes in mitochondrial dynamics. These conditions impair the contribution of mitochondria to cellular bioenergy, increase ROS production, and may trigger unintended permeabilisation of the mitochondrial membrane, leading to inflammation and cell death [2]. Numerous studies in humans and animal models have shown that IR is associated with reduced mitochondrial mass or oxidative function in insulin-sensitive tissues. On the one hand, altered mitochondrial function reduces oxidative capacity and β-oxidation, induces lipid species accumulation, and ultimately alters insulin signalling. On the other hand, mitochondria-generated ROS directly or indirectly impair insulin signalling through inflammasome activation [136]. However, it was found that mitochondrial phagocytosis can remove dysfunctional mitochondria through the PINK1/Parkin pathway as a way to reduce ROS damage to the insulin signalling pathway. Meanwhile, inflammatory factor production can be reduced during autophagy, improving insulin sensitivity in adipose tissue and the liver.

In experimental studies of animal models of ageing, such as nematodes, Drosophila, or mice, it has been observed that reduced levels of insulin or insulin signalling promote longevity and that there is a strong genetic basis for the pro-ageing effects of the insulin signalling pathway. Similarly, in humans, both hyperinsulinemia and concomitant IR are associated with an elevated risk of age-related diseases [137]. Although several studies have demonstrated that down-regulation of the insulin signalling pathway has a life-extending effect in model organisms, the mechanism of interaction between IR and β-cell functional decline during human ageing and its clinical significance still need to be further elucidated. The key questions are: is the ageing-associated decline in insulin sensitivity associated with IR, and what are the dynamic changes in β-cell function with age? A US population-based study showed that IR was significantly associated with cellular senescence, and the association remained significant after controlling for multiple demographic and lifestyle covariates simultaneously [138]. It is also worth noting that peripheral IR may also exacerbate ageing. Ropelle et al. [139] showed that ageing in mice was associated with increased iNOS expression of insulin receptor IRS-1 and AKT/PKB with increased S-nitrosylation in skeletal muscle as well as IR. Kahn’s team [140] discovered that when matching participants based on body mass index (BMI) and insulin sensitivity, healthy older men aged 61 to 82 years exhibited a significant 46% reduction in their acute insulin secretory response to intravenous glucose stimulation. Despite this, their baseline insulin levels were similar to those of younger controls aged 24 to 31 years. This suggests that ageing may selectively impair the rapid secretory capacity of β-cells. This finding aligns with the clinical data from Ahren and Pacini [141]. In older people subjects aged 63 years, who were matched based on an Oral Glucose Tolerance Test (OGTT), there was no difference in insulin secretion and insulin sensitivity during the first phase. However, in the second (delayed-phase) response, there was a sharp 56% decrease in insulin secretion. This indicates that the impairment of β-cell function due to ageing may have a specific sequence over time. Although several studies have demonstrated that down-regulation of the insulin signalling pathway has a life-extending effect in model organisms, the mechanism of interaction between IR and β-cell functional decline during human ageing and its clinical significance still need to be further elucidated.

## 4. Atherosclerosis

Atherosclerosis is a lipid-driven, multifocal, negatively combustible immunoinflammatory disease that occurs in medium- and large-sized arteries. The underlying pathologic change is the formation of plaques on the intimal surface of the artery, with lipid streaks, fibrous plaques, and atheromatous plaques. Continued aggravation of the lesion may result in secondary lesions such as calcification, atheromatous ulcer formation, thrombosis, and intraplaque haemorrhage. The aetiology and pathogenesis of atherosclerosis are complex and have not yet been fully elucidated. However, the main risk factors are hyperlipidemia, hypertension and heavy smoking, as well as diabetes mellitus, obesity, immune damage and genetic factors.

### 4.1. Mechanisms of Atherosclerosis

The thickening of the arterial wall associated with atherosclerosis and the consequences it produces make it a very complex disease entity with many mechanisms that contribute to its production [142]. Atherosclerosis is thought to be caused by a variety of factors, including genetic and environmental factors. There are many known risk factors for it, including hypercholesterolemia, hypertension, diabetes, kidney disease, and smoking. Chronic arterial inflammation caused by these risk factors can lead to intimal plaque formation [143].

Russell Ross first suggested in 1999 that atherosclerosis may be an inflammatory disease. He suggested that intimal infiltration, modification of plasma-derived lipoproteins and their primary uptake by macrophages lead to the formation of lipid-filled foam cells, which are the beginning of atherosclerotic lesions. Inflammation begins with inflammatory vesicles, which are innate immune signalling complexes that are important regulators of IL-1 family cytokine production in atherosclerosis and contribute to the vascular inflammatory response that drives the development and progression of atherosclerosis [144]. In turn, IL-1 family cytokines are major contributors to vascular inflammation and play an important role in the initiation and progression of atherogenesis. Macrophages, which are essential mediators and coordinators of various chronic inflammatory conditions, also play a crucial role in the formation and progression of atherosclerosis [145]. It has been shown that under pathological conditions in the atherosclerotic microenvironment, macrophages acquire both pro- and anti-inflammatory phenotypes, characterised by the release of the corresponding molecules. Under chronic low-grade inflammatory conditions, macrophages exhibit catabolism, degrading and thinning the fibrous cap and thus the fibrous atheromatous plaque. In turn, chronic low-grade inflammation of the vessel wall induces the accumulation of senescent cells. Under such conditions, the inflammatory microenvironment acquires a senescence-associated secretory phenotype that plays a crucial role in the development of early vascular senescence syndromes and cardiac senescence [146].

The endothelial response plays a crucial role in the inflammatory response. Research has demonstrated that, under normal physiological conditions, the vascular endothelium exhibits antithrombotic, anti-inflammatory, and vasoactive properties. These properties regulate the permeability of the vessel wall, allowing it to manage the passage of bioactive molecules in the bloodstream and maintain vascular tone by balancing the release of vasodilators and vasoconstrictors. The development of endothelial dysfunction, characterised by pro-inflammatory and vasospastic responses, leads to an abnormal increase in vascular permeability and a reduction in the bioavailability of atheroprotective nitric oxide, resulting in atherogenic formations [147,148,149,150,151].

**Figure 4:**
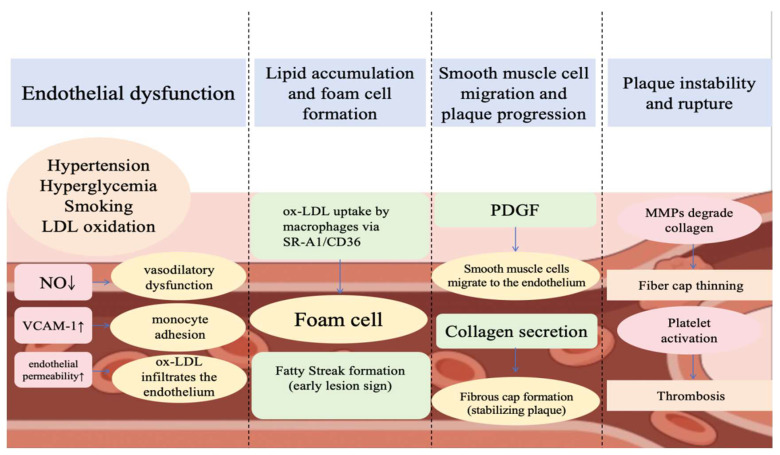
Diagram of atherosclerotic mechanisms This schematic illustrates the key mechanisms linking endothelial injury to plaque formation: (1) Risk factors (hypertension, hyperglycemia, smoking, and LDL oxidation) induce endothelial dysfunction, characterized by reduced NO bioavailability (leading to vasodilatory impairment, increased monocyte adhesion, and ox-LDL infiltration); (2) Oxidized LDL (ox-LDL) uptake by macrophages via scavenger receptors (SR-A1/CD36) drives foam cell formation; (3) Platelet-derived growth factor (PDGF) mediates smooth muscle cell migration, collagen secretion, and fibrous cap formation, while also promoting platelet activation and thrombosis risk. Upregulation of VCAM-1 further exacerbates endothelial permeability and leukocyte recruitment. The upward and downward arrows indicate increase and decrease, respectively.

One of the key events in the formation of atherosclerosis is the cumulative oxidation of LDL that accumulates within plaques [152,153]. LDL is the main carrier of cholesterol in cells, and during oxidative stress, LDL is modified to Ox-LDL [154]. Ox-LDL is a more potent pro-atherosclerotic mediator than native LDL. It is formed by the activation of native, unmodified LDL through the initiation of lipid peroxidation by several ROS. Ox-LDL contains varying proportions of a variety of toxic oxidized lipids, whose toxic constituents may include aldehydes, sterols, lipid peroxides. The interaction between Ox-LDL and other risk factors stiffens and narrows the arterial lumen, leading to disturbed blood flow [155]. It inhibits normal vasorelaxation by reducing NO production and enters macrophages to promote foam cell formation. Foam cells are particularly prone to secreting inflammatory cytokines and undergoing apoptosis. Relevant studies have shown that in atherosclerosis, Ox-LDL can modulate macrophage polarisation through cell signalling, metabolic reprogramming, epigenetics, and intercellular communication to promote vessel wall inflammation and exacerbate the progression of atherosclerosis. Currently, antidiabetic drugs have been clinically found to reduce glucose-induced oxidative stress by lowering blood glucose levels, thereby preventing the conversion of LDL to Ox-LDL, which accelerates the atherosclerotic process [156]. In addition, PUFAs have been shown to have significant atheroprotective properties, with linoleic acid inhibiting the expression of pro-inflammatory genes in macrophages and reducing the progression of atherosclerosis by inactivating NF-κB, CCL2, and COX-2 via the PPARγ receptor [157]. PUFAs modulate the atherogenic effects of saturated fatty acids, such as palmitate-induced expression of the oxidised LDL-1 (LOX1) lectin-like receptor [158]. Atherosclerosis-related mechanisms are shown in Figure 4 [146,147,148,149,150,151].

### 4.2. The Relationship Between Aging and Atherosclerosis

Although long-term trends in age-standardised cardiovascular disease mortality and morbidity indicate a substantial decline in the burden of cardiovascular disease, the impact of population growth and ageing has resulted in a continued increase in the absolute number of people with cardiovascular disease [159]. Numerous studies have found that a variety of senescent cells in the vasculature are closely associated with changes in atherosclerotic pathophysiology, while SASPs secreted by senescent cells contribute to the progression and imbalance of atherosclerotic plaques [160]. Dysfunctional senescent endothelial cells play a key role in aspects of vascular morphology and physiology, and senescent endothelial cells are frequently found in atherosclerotic plaques of older patients [161]. The accumulation of senescent endothelial cells in the aortic wall further induces endothelium-dependent dilatation dysfunction, leading to fibrinolysis and increased permeability. Decreased NO production and increased ET-1 release in senescent endothelial cells compared with normal endothelial cells promote vasoconstriction and accelerate late thrombosis. In addition, telomere shortening contributes to cellular senescence, and in atherosclerotic lesions, telomere shortening of endothelial cells is observed and is accompanied by the appearance of senescence markers. Structurally, as blood vessels age, the arterial lumen gradually expands, different degrees of thickening and calcification occur in the intima and media, and the number of capillaries decreases, further exacerbating the progression of atherosclerosis. In addition, it has been shown that vascular smooth muscle cells and collagen constitute the vascular elastic layer. They maintain vascular tone and blood pressure and participate in vascular remodelling. In ageing arteries, vascular smooth muscle cells migrate from the mid-membrane to the subendothelium and are deposited in the extracellular matrix. They encapsulate phagocytosed lipid macrophages and other inflammatory cells, forming fibrous plaques and inducing atherosclerosis.

Another important reason for the development of atherosclerosis is the development of chronic inflammation. With the ageing process, the levels of pro-inflammatory cytokines increase significantly in older people. The ageing vascular lining exhibits both oxidative stress and inflammatory activation. Reactive oxygen species activate inflammatory signalling pathways, including NF-ĸB, and produce pro-inflammatory cytokines by regulating the paracrine effects of endothelial cells, leading to atherosclerosis. Senescent endothelial cells secrete inflammatory mediators and ROS. Concomitantly, retention of Ox-LDL in the arterial intima, monocyte adhesion to the endothelium, activation of NF-κB signalling, and production of extracellular vesicles are potent promoters of SASP-mediated senescence, which may play a role in atherosclerotic plaque development [162].

### 4.3. Relationship Between IR and Atherosclerosis

IR is a major cause of atherosclerosis and cardiovascular disease. IR leads to a chronic low-grade inflammatory state in adipose tissue and other metabolically active tissues, causing these tissues to release a variety of inflammatory mediators, including cytokines, chemokines, and adhesion molecules. These mediators attract and activate inflammatory cells, such as monocytes and macrophages, to infiltrate the vessel wall and release additional inflammatory mediators and growth factors. This stimulates the proliferation, migration and phenotypic transformation of vascular smooth muscle cells, leading to the formation and progression of atherosclerotic plaques [163]. Meanwhile, related studies have shown that oxidative stress promotes the development of cardiac IR, diabetic cardiomyopathy and heart failure. With the development of hyperglycemia and IR, the influx of NAD+ and FAD+ into the mitochondria is increased, which leads to hyperpolarization of the inner membrane. Nicotinamide adenine dinucleotide phosphate (NADP) is another important source of ROS in cardiomyocytes and can lead to excessive accumulation of ROS [164]. Simultaneous hyperglycemia and IR inhibit Nrf2 expression and activity via Erk1/2-mediated pathways, which suppresses myocardial defense and stimulates the development of ROS and calcium overload-related changes [165].

In a study conducted by Yi et al. [166], IR was evaluated using the estimated glucose handling rate. The study aimed to examine the relationship between this estimated glucose handling rate, which serves as a surrogate indicator of IR, and the incidence of atherosclerotic cardiovascular disease. The study found a significant inverse relationship between estimated glucose handling rate and atherosclerotic cardiovascular disease incidence. The importance of controlling in mitigating the risk of atherosclerotic cardiovascular disease and improving cardiovascular outcomes in different populations was also emphasised. In addition, the association of surrogate indices of IR, including the homeostatic model assessment of IR, the triglyceride–glucose index and its related indices, with the risk of atherosclerotic cardiovascular disease incidence in the general population has been extensively studied [167,168]. The results of most studies suggest that an elevated homeostatic model assessment of IR or triglyceride–glucose index and its related indices lead to an increased risk of atherosclerotic cardiovascular disease or coronary heart disease severity [169,170].

In addition, IR leads to impairment of the insulin signalling pathway and compensatory secretion of more insulin by the pancreas. High insulin levels directly stimulate the proliferation of vascular smooth muscle cells and promote arterial wall thickening. IR also increases the release of free fatty acids, leading to increased hepatic synthesis of very low-density lipoproteins while decreasing high-density lipoproteins. Smaller and denser LDL is more easily oxidised and phagocytosed by macrophages to form foam cells, which are deposited in the vessel wall and accelerate the process of atherosclerosis.

## 5. Discussion

This review examines changes in various bodily systems during ageing and their underlying mechanisms, with a particular focus on the pathogenesis of two representative age-related diseases: IR and atherosclerosis. By synthesising key research across the fields of ageing, metabolism, and cardiovascular disease, it reveals how ageing exacerbates IR, thereby driving the progression of atherosclerosis. Discussions of several mechanistic theories of ageing reveal disagreements and controversies surrounding each. For instance, within the somatic mutation theory, it has been observed that the accumulation of mutations may not necessarily originate from cell division. This finding underscores that ageing is a complex process involving the synergistic action of multiple mechanisms.

In examining two representative age-related diseases, this review elucidates the linkages between ageing and these conditions, as well as their shared molecular pathways—including inflammatory pathways, oxidative stress pathways, and mitochondrial dysfunction. It also systematically summarises the relationship between IR and atherosclerosis, analysing specific measures to effectively delay ageing, improve IR, and prevent the progression of atherosclerosis. This provides important theoretical support for the early prevention of chronic diseases in the elderly. However, the common pathways of ageing-related diseases may simultaneously participate in multiple physiological and pathological processes. Targeting these pathways for intervention may induce side effects, potentially disrupting normal signal transduction.

Research indicates that chronic inflammation is significantly more prevalent and severe among older adults with visceral obesity, metabolic syndrome, type 2 diabetes, cardiovascular disease, and neurodegenerative disorders [171]. During the ageing process, circulating levels of IL-6, TNF-α, IL-1, and other pro-inflammatory markers increase with age, even in healthy individuals. Elevated levels of inflammatory markers lead to persistent activation of the immune system, accelerating the ageing process and activating multiple inflammatory signalling pathways. These inflammatory factors directly interfere with insulin signalling pathways during ageing. For example, TNF-α inhibits the tyrosine phosphorylation of IRS, a crucial step in insulin signalling. The activation of the downstream PI3K-Akt pathway leads to the phosphorylation of multiple downstream target proteins, resulting in diverse biological effects.

IR contributes to the development of multiple diseases, with compensatory hyperinsulinemia serving as the core pathophysiological link connecting metabolic disorders to cardiovascular disease. When the PI3K-Akt pathway is inhibited, it reduces the production of nitric oxide (NO) by vascular endothelial cells. NO is a potent vasodilator that lowers blood pressure and suppresses inflammation and oxidative stress. Concurrently, hyperinsulinemia increases secretion of endothelin-1 (ET-1), a potent vasoconstrictor. Disruption of the balance between vasodilation and vasoconstriction leads to sustained vascular constriction and elevated blood pressure, thereby accelerating the progression of atherosclerosis.

Through investigation into the mechanisms linking ageing, IR, and atherosclerosis, it has been established that developing and implementing treatment strategies to predict IR and atherosclerosis in elderly patients is of paramount importance. Relevant studies indicate that the triglyceride–glucose index (TyG) can serve as a risk biomarker for major adverse cardiovascular events [172]. The TyG index is a composite indicator that assesses IR by analysing fasting triglyceride (TG) and fasting glucose (FG) levels. Lipoproteins rich in triglycerides can significantly influence the development of atherosclerosis. Studies reveal that individuals with elevated TyG indices exhibit a higher proportion of patients at increased risk for cardiovascular events, along with markedly higher incidence rates of metabolic disorders, type 2 diabetes, and non-alcoholic fatty liver disease [173].

We have found that most current research is based on animal models or in vitro cell experiments, lacking long-term, large-scale intervention studies conducted on human populations. Due to differences between the complex physiological environment of humans and animals, the conclusions drawn are inconsistent, leading to discrepancies and controversies. For instance, experiments using mice as subjects reveal significant differences in metabolic rates, lifespan, and immune responses compared to humans. This disparity makes it challenging to directly translate specific research findings into clinical applications, complicating the clinical translation of potential therapeutic targets. Furthermore, variations in genetic backgrounds, lifestyles, and environmental factors among individuals lead to differences in ageing rates and disease risks, making universal therapies difficult to apply to all populations.

Current research utilises organoids or organ-on-a-chip derived from human pluripotent stem cells to simulate human ageing, thereby mitigating the limitations of animal models to some extent. Concurrently, real-time monitoring technologies based on artificial intelligence and big data platforms enable the development of personalised anti-ageing strategies tailored to individual genomes, lifestyles, and clinical data, facilitating dynamic interventions. Furthermore, we can intensify the functional exploration of existing drugs, such as insulin sensitisers—metformin, thiazolidinediones, and SGLT2 inhibitors—to investigate whether cardiovascular protection can be achieved by modulating specific pathways (e.g., inflammation suppression). Targeted research should also focus on specific points within the ageing-IR-atherosclerosis axis. Future studies aim to deepen our understanding of ageing-related diseases and mechanisms, providing a robust theoretical foundation for drug development and therapeutic strategies. This will enable the delay, prevention, or mitigation of multiple age-related conditions, ultimately achieving ageing prevention, slowing down the ageing process, and potentially even reversing biological ageing.

## 6. Conclusions

In summary, this review systematically constructs a unified theoretical framework that integrates fragmented knowledge from gerontology, metabolic medicine, and cardiovascular medicine. It explores the relationship among ageing, IR, and atherosclerosis, concluding that the core mechanism driving their synergistic progression may be a vicious cycle induced by chronic low-grade inflammation, oxidative stress, and epigenetic alterations. This review synthesises findings across disciplines, providing a foundational theoretical basis for future mechanistic investigations and clinical translation. We further propose that future studies employ genetic screening to identify high-risk elderly populations, prioritising clinical trials that combine senescent cell clearance agents with anti-inflammatory drugs for this cohort. Furthermore, multi-omics technologies should be employed to identify novel biomarkers within the ageing-IR-atherosclerosis axis, enabling targeted research on these emerging markers. Future efforts will uncover feasible strategies to break the vicious cycle among these three factors and effectively extend healthy lifespan.

## Figures and Tables

**Table 1:** Mechanisms of Ageing Doctrine.

Doctrines	Mechanisms	Direction of Intervention
Somatic cell mutation theory	Oxidative damage, Replication errors, Spontaneous hydrolysis—DNA damage and mutation accumulation—Functional gene inactivation, Mitochondrial DNA mutations, Epigenetic disorders—Fixing system degradation—Reduced repair enzyme activity, Telomere depletion	Activate pathways, such as AMPK/SIRT1, to indirectly enhance cellular stress resistance and DNA repair capacity. Removal of senescent cells with high mutational load Reduction in ROS-induced mutations
Free radical theory	Lipid peroxidation—Attacking unsaturated fatty acids in cell membranes—Decreased membrane fluidity and increased permeability Protein oxidation—Disrupts the activity of enzymes, receptors, and other proteins—Formation of protein cross-links or aggregates DNA damage—Causes base modification, strand breaks—Mutation or apoptosis Mitochondrial damage—Damage to mitochondrial DNA—Energy metabolism disorder	Reducing free radical production: improving mitochondrial function, lifestyle modifications Enhancement of antioxidant defense system: supplementation of exogenous antioxidants, activation of endogenous antioxidant pathways Repairing oxidative damage: removing damaged molecules and enhancing DNA repair Regulation of intestinal flora Related gene therapy
Immunological theory	Thymus atrophy—Decreased T-cell production—Memory T cell accumulation—Decreased immune response capacity Decreased B-cell function—Reduced antibody diversity—Diminished vaccine response Decreased NK cell activity—Decreased clearance of viral infections and tumour cells Abnormal macrophage and dendritic cell function—Decreased antigen presentation capacity and imbalance in inflammatory regulation Cellular senescence—Senescent cells secrete pro-inflammatory factors (SASP, IL-6, TNF-α) Mitochondrial dysfunction—Increased activation of NLRP3 inflammatory vesicles by ROS—IL-1β release Intestinal flora imbalance—Age-related changes in flora—Endotoxin (LPS) into the blood—Activation of the TLR4-NF-κB pathway Autoantigen accumulation—Protein misfolding and oxidative damage—Triggering an autoimmune response	Enhancement of immune system function: thymus regeneration, immune cell therapy, vaccine optimization Suppression of chronic inflammation: removal of senescent cells, application of anti-inflammatory drugs Regulates intestinal flora Metabolic and nutritional interventions
Telomere theory	Telomere shortening to critical length (Hayflick limit)—Activation of the p53/p16INK4a pathway—Cell cycle arrest Loss of telomere function—Chromosome fusion, breakage—Increased risk of cancer promotion Shortening of telomeres in hematopoietic stem cells, intestinal epithelial stem cells—Decreased tissue regeneration Secretion of SASP by senescent cells—Chronic inflammation	Activation of telomerase: TERT gene therapy, small molecule activators Antioxidant and telomere protection: NAD+ enhancers, telomere-targeting antioxidants, dietary interventions Removal of senescent cells with shortened telomeres Improvement of lifestyle Gene editing and cell therapy: CRISPR gene editing, iPSC technology
Neuroendocrine theory	Hypothalamic-pituitary-target gland axis hypofunction—(1) Decrease in hypothalamic growth hormone-releasing hormone (GHRH) and senescence of pituitary GH-secreting cells—Muscle loss, Fat accumulation, Skin thinning, Decreased tissue repair (2) Cortisol dysregulation, decreased DHEA—Promotes inflammation and muscle breakdown, affects immune and cognitive functions (3) Decrease in sex hormones—Osteoporosis, Reduced vascular elasticity, Muscle loss, Cognitive decline (4) Decreased melatonin—Pineal gland failure—Sleep disorders, decreased antioxidant capacity Dysregulation of hypothalamic centres of ageing and metabolic regulation—(1) Hypothalamic neural stem cell depletion—Affects body temperature, appetite, and circadian rhythm regulation (2) Leptin and insulin resistance—Obesity and metabolic syndrome Neuroendocrine-immune interactions—Chronic inflammation—Cortisol resistance, Decreased GH/IGF-1	Hormone replacement therapy: growth hormone, sex hormones, DHEA supplementation Targeting hypothalamic function modulation: senolytics remove senescent cells, NAD+ enhancers, rapamycin Melatonin and circadian regulation: exogenous melatonin, light therapy Lifestyle and nutritional interventions: intermittent fasting, resistance training, Mediterranean diet Emerging therapies: stem cell therapy, gene therapy

**Table 2:** Summary of studies on the doctrine of ageing-related mechanisms.

Author	Type of Experiment	Experimental Conditions	Theory	Result
Cagan [21]	animal experimentation	Isolation of 208 individual intestinal crypts from 56 individuals of 16 species and study of somatic mutations using standard whole genome sequencing	somatic cell mutation theory	Significant accumulation of somatic mutations with age
Garger [22]	database analysis	Obtaining data from public databases to construct phylogenetic tree sets for linear modeling analysis	somatic cell mutation theory	Somatic cell mutation rate is highly correlated with longevity
Abascal [23]	cellular experiment	Development of nano-rate sequencing (NanoSeq) to achieve an error rate of less than 5 per billion base pairs in a single DNA molecule in a population of cells	somatic cell mutation theory	Somatic cell mutation rate is independent of cell division rate
Robinson [25]	clinical research	Fourteen individuals aged between 17 and 72 years each carried one of four different germline extracellular ribozyme structural domain mutations in POLE or POLD1	somatic cell mutation theory	Significantly elevated mutation load in somatic cells with unique mutational features that do not exhibit premature senescence
Robert [30]	animal experimentation	Metabolic rates, locomotor performance, cellular metabolic rates, and oxidative stress potentials were measured in six snake species with varying lifespans	free radical theory	Short-lived species exhibit lower ROS production
Ali [31]	animal experimentation	Exploring the association between oxidative stress and sex-specific aging in C57BL6 mice, ROS were measured in young and old mice by confocal imaging of DHE oxidation in the brain and EPR spectroscopy of isolated brain mitochondria	free radical theory	Sex differences in free radical homeostasis as a determinant of longevity
Wang [48]	meta-analysis	Twenty-five studies were included, 21 used quantitative PCR and 4 used SB	telomere theory	Telomere shortening is associated with increased all-cause mortality in the general population
Ye [49]	meta-analysis	In total, 236 studies comprising 341 samples across 720,078 subjects were included as cross-sectional samples, and 46 studies comprising 73 samples across 22,941 subjects were included as longitudinal samples	telomere theory	Telomere length decreases with actual age
Sanchez [51]	cohort study	The potential of DTM as a clinical research tool was demonstrated by examining telomere length distributions in cross-sections of 63 healthy and diseased human samples	telomere theory	Distribution scores for shorter telomere composition increase with age
Rosen [55]	cohort study	Using a self-rating questionnaire, the results were compared with those of 86 controls matched for age, sex, marital status, and socioeconomic class	neuroendocrine theory	Growth hormone therapy can lead to increased energy and emotional stability in older adults.

**Table 3:** Measures to delay ageing.

Methods	Measures
Targeting Senescent Cells	Remove senescent cells: Senolytics (senescent cell lyser)—Dasatinib + Quercetin, Fisetin, Navitoclax (ABT-263) SASP inhibition: Senomorphics (Phenotypic modulators of senescent cells)—Rapamycin, JAK inhibitors, metformin Enhancement of the immune system: CAR-T cell therapy, development of senescent cell-specific antigen grafts Gene cell therapy: p16INK4a modulation, stem cell therapy
Delayed telomere shortening	Activation of telomerase: TERT mRNA therapy, small molecule telomerase activators Telomere protection: application of antioxidants, NAD+ enhancers Improve lifestyle to delay telomere shortening
Metabolic intervention	Optimizing Energy Metabolism: NAD+ Supplements Regulation of nutrient-sensing pathways: inhibition of mTOR overactivation, activation of AMPK, optimization of insulin sensitivity Reduction in advanced glycation end-products (AGEs): low AGEs diet, AGEs blockers Promoting metabolic waste removal: autophagy and detoxification
Gut microbial interventions	Supplementation with prebiotics, probiotics Fecal Microbiota Transplantation (FMT)
Lifestyle interventions	Calorie restriction and intermittent diet, proper exercise, better sleep, stress relief

## Data Availability

All data available.

## Abbreviations

The following abbreviations are used in this manuscript:

IR	Insulin resistance
LDL	Low-density lipoproteins
VLDL	Very-low-density lipoprotein
HDL	High-density lipoprotein
LPO	Lipid peroxides
ROS	Reactive oxygen species
IGF-1	Serum Insulin-like Growth Factor 1
SASP	Senescence-associated secretory phenotype
PIP2	Phosphatidylinositol 4,5-diphosphate
PIP3	Phosphatidylinositol (3,4,5)-trisphosphate
GLP-1	Glucagon-like peptide 1 receptor
SGLT-2	Sodium-glucose co-transporter protein
PUFAs	Polyunsaturated fatty acid
O_2_	Oxygen
IRS2	Insulin Receptor Substrate 2
NAD+	Nicotinamide adenine dinucleotide
NAMPT	Nicotinamide phosphoribosyltransferase
SCN	Suprachiasmatic Nucleus
FFAs	Free Fatty Acids
NADP	Nicotinamide adenine dinucleotide phosphate
NO	Nitric oxide
ET-1	Endothelin-1
TyG	Triglyceride–glucose index
AMPK	Adenosine Monophosphate-activated Protein Kinase
TP53	Tumor Protein p53
KRAS	Kirsten Rat Sarcoma Viral Oncogene Homolog
O_2_•^−^	Superoxide Anion
H_2_O_2_	Hydrogen Peroxide
·OH	Hydroxyl Radical
NO·	Nitric Oxide
IL-6	Interleukin-6
TNF-α	Tumor Necrosis Factor Alpha
NLRP3	NOD-, LRR- and Pyrin Domain-Containing Protein 3
IL-1β	Interleukin-1 Beta
TLR4	Toll-Like Receptor 4
NF-κB	Nuclear Factor Kappa-Light-Chain-Enhancer of Activated B Cells
LPS	Lipopolysaccharide
CAR-T	Chimeric Antigen Receptor T-Cell Therapy
TERT	Telomerase Reverse Transcriptase
p16INK4a	Cyclin-Dependent Kinase Inhibitor 2A
mTOR	Mechanistic Target of Rapamycin
FMT	Fecal Microbiota Transplantation
Shc	Src Homology and Collagen Homology
Grb2	Growth Factor Receptor-Bound Protein 2
PI3K	Phosphoinositide 3-Kinase
Ras	Rat Sarcoma virus
Raf	Rapidly Accelerated Fibrosarcoma
MEK	Mitogen-Activated Protein Kinase Kinase
ERK1/2	Extracellular Signal-Regulated Kinase 1 and 2
GSK3β	Glycogen Synthase Kinase 3 Beta
FOXO1	Forkhead box protein O1
AS160	Akt Substrate of 160 kDa
CREB	CAMP Response Element-Binding protein
mTORC1	Mammalian Target Of Rapamycin Complex 1
PEPCK	Phosphoenolpyruvate carboxykinase
G6Pase	Glucose-6-Phosphatase
GLUT4	Glucose Transporter type 4
SREBP1c	Sterol Regulatory Element-Binding Protein 1c
VCAM-1	Vascular Cell Adhesion Molecule-1
PDGF	Platelet-Derived Growth Factor
ox-LDL	Oxidized Low-Density Lipoprotein
